# Transparency and training in peer review: Discussing the contributions of early-career researchers to the review process

**DOI:** 10.1038/s42003-021-02646-5

**Published:** 2021-09-24

## Abstract

This year’s theme for Peer Review Week is “Identity”, with a focus on promoting equity in peer review practices and recognizing how personal identity can influence the process. While many researchers may involve trainees with their reviews, not all will acknowledge the contributions made by these early-career researchers or request that journals provide them with direct recognition. In this Q&A, we asked pairs of faculty and post-doctoral fellows who previously co-reviewed manuscripts at *Communications Biology* to reflect on their experiences with peer review, and the importance of including and recognizing early-career researchers as part of this process.

**Dr. Stefanie Robel** is an Assistant Professor at the Fralin Biomedical Research Institute at the Virginia Tech Carilion School of Neuroscience. She received her Ph.D. in neurobiology from Ludwig-Maximilian University and Helmholtz Zentrum München and was a post-doctoral fellow at the University of Alabama at Birmingham before joining the faculty at Virginia Tech in 2015.

**Figure:**
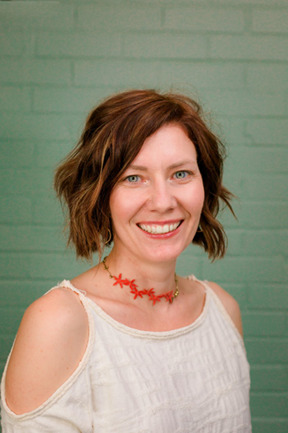
Stefanie Robel

**Dr. Carmen Muñoz-Ballester** received her Ph.D. from Institute of Biomedicine of Valencia before joining the Robel lab as a post-doctoral fellow. In the future, she would like to lead her own lab with a focus on the molecular basis of sex differences in cognitive decline and aging.

**Figure:**
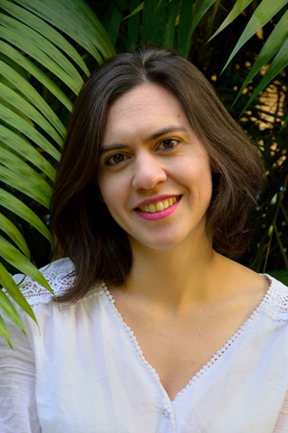
Carmen Muñoz-Ballester

**Dr. Yan Zhu** is a Principal Investigator at the Institute of Biophysics at the Chinese Academy of Sciences. He received his Ph.D. in molecular and cell biology from Washington University in St. Louis and completed a post-doctoral fellowship at the University of California, Los Angeles, before starting his lab at the Chinese Academy of Sciences in 2009.

**Figure:**
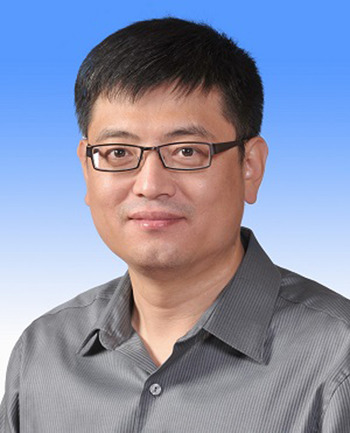
Yan Zhu

**Dr. Shaowei Hu** received his Ph.D. in neuroscience from the Chinese Academy of Sciences in 2017, and is currently a post-doctoral fellow in the Zhu lab.

**Figure:**
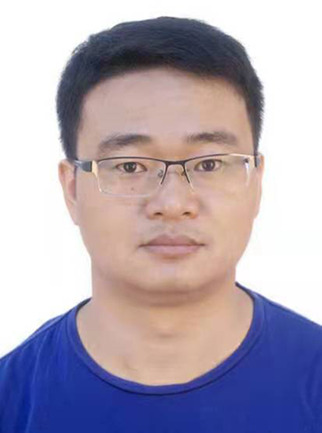
Shaowei Hu

Dr. Robel and Dr. Muñoz-Ballester, please tell us about your research interests.

**Stefanie Robel (SR):** Proper brain function is mediated by neurons and shaped by glial cells. Astrocytes are glial cells in the brain that have many roles during development and in the mature brain. Our lab studies the fascinating changes that astrocytes undergo in neurological disease or when the brain is injured by mild traumatic brain injury or concussion.

We ask how these changes affect astrocyte functions: (1) At the synapse, where astrocytes are responsible for maintaining ion and neurotransmitter homeostasis. (2) At the network level - Do local changes in astrocyte function affect neuronal network function and behavior? (3) At the vasculature, where astrocytes contribute to the maintenance of the blood-brain barrier and regulation of blood flow. We study how cellular changes affect astrocyte physiology in the context of brain injury or disease and if these may contribute to neurodegeneration and dementia.

**Carmen Muñoz-Ballester (CMB):** I decided to do my post-doc in this laboratory because I am fascinated by astrocytes, glial cells in the brain that were thought to be mere structural support for neurons for a long time, but we now know are essential to maintain the healthy functions in the brain. I am also interested in the response of the brain to injury and other physiological processes like aging, and astrocytes are key in the adaptation of the brain to these new conditions. More specifically, I am studying why females respond differently to brain injury and aging than males and the role that astrocytes are playing in these contexts.

Dr. Zhu and Dr. Hu, please tell us a little about your research.

**Yan Zhu (YZ):** I am interested in the neural basis of desires. Using *Drosophila* as a model organism, I investigate the neural circuits regulating the basic processes of thirst for water, appetite, mating, and aggression.

**Shaowei Hu (SH):** My research interests include: (1) the neural and molecular basis of innate behaviors like aggressive behavior and courtship behavior; (2) gene therapy for inherited diseases like deafness.

Why is it important to include trainees in the peer review process and provide them with direct recognition for their involvement?

**SR:** From my perspective, it is critically important to extend the training that mentees receive, especially at the post-doc level, to all activities that a principal investigator is responsible for so that they can make informed career decisions. This includes aspects of team leadership and management, as well as communication with and service to the research community. The publishing process can be difficult to navigate without insight into all aspects of it. In my lab we spend time discussing the different roles that researchers take when participating in the process. Yet, a direct experience is worth a thousand words, which is why I ask mentees to participate in the peer review process. Additionally, post-doctoral fellows in my lab are often closer to the technical aspects of the work or have stronger expertise in parts of the work that is being evaluated. Working on a peer review together allows me to train mentees in a systematic approach to evaluating manuscripts, as well as in writing a review that ensures scientific integrity and is genuinely helpful to the authors. I put emphasis on striking a professional tone that lays out our perspective about the quality of the manuscript, strengths and weaknesses without coming across as harsh or judgmental. Because a high-quality review takes considerable effort and time, especially when mentees who have not yet reviewed many manuscripts, it is important to recognize the effort and involvement. It is also crucial to the transparency of the peer review process to disclose who reviewed the manuscript. Peer review is confidential and all people involved in the process should be on record with the journal. In addition, this creates a record that establishes a junior researcher as competent reviewer and increases the diversity of the reviewer pool.

**YZ:** Reviewing manuscripts is an important part of the training process for a trainee who would later become an independent researcher. The trainees deserve full recognition for their time, efforts, and intellectual contributions to the reviewing process.

**SH:** Most of the trainees will be independent investigators one day, so they must learn how to judge unpublished work from other groups. Including trainees in the process will ensure that peer review is a self-sustained process with constantly high standards over time.

**CMB:** In my opinion, a mentor is much more than a person who guides you through your science but someone who helps you to grow as a scientist in every aspect necessary to succeed in your career. In my case, I want to lead an independent research program, and being able to be a good reviewer who helps the authors, the journal, and the scientific community to have reliable and solid manuscripts is a contribution that is expected from a good academic. While I could learn this skill on my own, it was much better for me to have the exposure to the peer review system with my mentor, not feeling disoriented and preparing me for future independent peer reviews. On the other hand, I also put my time and effort to do a good job and, as in every other contribution to science, it is ethical to acknowledge this work to the person who did it. Just like a person who contributed to a paper should be an author, everyone who contributes to peer review should be named a reviewer. You did the work; you get the recognition. I just wish more journals informed principal investigators and reviewers in general about the possibility of co-reviewing manuscripts with trainees.

What has been your main takeaway from the peer review experience, and why is peer review important?

**YZ:** Peer review critically evaluates a manuscript for its merit. More importantly, the process gives authors opportunities to obtain constructive suggestions, which are directly related to their research, from the experts in the field. Because of this, I had much improved manuscripts at the end of the process. Additionally, I observed quite diverse perspectives of thinking in the reviewers.

**CMB:** Peer review is a system that allows the scientific community to improve a scientific publication. Reviewers bring a new pair of eyes on the argument, helping to have more clarity and to identify blind spots. While I do not think improving a manuscript is necessary the work of a reviewer, I truly believe that it is our responsibility as scientists to make sure that any manuscript has certain standards of quality and reliability.

Being on the other side of the peer reviewer process definitely gave me a new perspective. I understood better how we, as authors, can many times assume what our readers know or go too far in our conclusions. However, an external reader, an expert in the field with more distance to the manuscript, can easily detect gaps or assumptions and request more clarification and, if needed, experimental proof of a statement. I think being a reviewer has given me more perspective to write my own manuscripts and improved my way of approaching my own science. Thus, I guess I could say that being a peer reviewer made me a better scientist.

*Interviews were conducted by Associate Editor George Inglis*.

